# Targeting Enterococci with Antimicrobial Activity against *Clostridium perfringens* from Poultry

**DOI:** 10.3390/antibiotics12020231

**Published:** 2023-01-21

**Authors:** Sara García-Vela, Laila Ben Said, Samira Soltani, Ramzi Guerbaa, Rosa Fernández-Fernández, Houssem Ben Yahia, Karim Ben Slama, Carmen Torres, Ismail Fliss

**Affiliations:** 1 Department of Food Science, University of Laval, Quebec, QC G1V 0A6, Canada; 2 Area of Biochemistry and Molecular Biology, OneHealth-UR Research Group, University of La Rioja, 26006 Logroño, Spain; 3 Laboratoire Bioressources, Environnement et Biotechnologie (LR22ES04), Institut Supérieur des Sciences Biologiques Appliquées de Tunis, Université de Tunis El Manar, Tunis 1006, Tunisia

**Keywords:** *Clostridium perfringens*, enterococci, enterocins, protective cultures, necrotic enteritis, whole genome sequencing

## Abstract

Necrotic enteritis (NE), caused by *Clostridium perfringens,* is an emerging issue in poultry farming. New approaches, other than antibiotics, are necessary to prevent NE development and the emergence of multidrug-resistant bacteria. Enterococci are commensal microorganisms that can produce enterocins, antimicrobial peptides with activities against pathogens, and could be excellent candidates for protective cultures. This study aimed to screen and characterize *Enterococcus* strains of poultry origin for their inhibitory activity against *C. perfringens*. In total, 251 *Enterococcus* strains of poultry origin plus five bacteriocin-producing (BP+) *E. durans* strains of other origins were screened for antimicrobial activity against the indicator *C. perfringens* X2967 strain using the “spot on the lawn” method. We detected thirty-two BP+ strains (eleven *Enterococcus faecium*, nine *E. gallinarum,* eight *E. faecalis,* three *E. durans*, and one *E. casseliflavus*). We further studied the antimicrobial activity of the supernatants of these 32 BP+ strains using agar well diffusion and microtitration against a collection of 20 *C. perfringens* strains. Twelve BP+ enterococci that were found to exhibit antimicrobial activity against *C. perfringens* were characterized using whole genome sequencing. Among these, *E. faecium* X2893 and X2906 were the most promising candidates for further studies as protective cultures for poultry farming. Both strains belong to the sequence type ST722, harbor the genes encoding for enterocin A and enterocin B, do not possess acquired resistance genes, do not carry plasmids, and present the *acm* gene, which is implicated in host colonization. Further research is needed to determine the utility of these strains as protective cultures.

## 1. Introduction

Antibiotic resistance is a serious public health concern that compromises the treatment of infections in humans and animals and is associated with the unnecessary prescription and/or misuse of antibiotics. Besides their clinical use in humans, antibiotics are also used in veterinary and animal farming. Antibiotics have also been extensively used as growth promoters in food-producing animals; however, even though this practice has been banned in Europe since 2006 [1] and also in several other countries, it is still allowed in some others [2]. This contributes to the increase and spread of antibiotic resistance, not only among pathogenic bacteria but also among commensal bacteria of the intestinal tract of humans and animals, which can lead to contamination via feces. Therefore, resistant bacteria can reach humans via the food chain and water or by contact with animals. For this reason, the World Health Organization (WHO) proposed to address this issue from a “One Health” perspective, establishing new alternatives to the use of antibiotics in livestock and agriculture [3].

*Clostridium perfringens* is associated with necrotic enteritis (NE) in poultry, and its prevalence has been increasing in countries that no longer use antibiotic growth promoters, which suggests that the same trend could also originate among other relevant pathogens [4]. NE caused by *C. perfringens* is one of the most common poultry diseases that cause substantial economic losses to the industry [5]. A prominent characteristic of NE is acute death, with mortality rates reaching 50%. Clinical signs include depression, dehydration, somnolence, ruffled feathers, diarrhea, and decreased feed consumption [6]. The subclinical form of this disease causes chronic damage to the intestinal mucosa of the chickens, leading to impaired nutrient absorption, reduced weight gain, and decreased overall performance. *Clostridium perfringens* is present in the intestines of healthy chickens but in a small proportion (less than 10^5^ CFU/g of the intestinal content); when its count increases, hen birds become susceptible to NE [1].

Antibiotic-resistant bacteria are prevalent in different environments and can be introduced into the food chain at various points. Poultry is a reservoir for antibiotic-resistant bacteria that can be transmitted to humans. The continuous and widespread use of antibiotics in farm animals may lead to changes in the bacterial environment, eliminating susceptible strains and allowing antimicrobial-resistant bacteria to survive and predominate. Furthermore, the continuous administration of antibiotics in feed may cause cross-resistance to therapeutic antimicrobial agents. Antimicrobial resistance and a gradual decrease in antibiotic sensitivity to anticoccidials in some strains of *Eimeria* spp. (a predisposing factor for NE) can exacerbate the presence of *C. perfringens* strains [7].

Protective cultures essentially consist of bacteria specifically selected for their ability to inhibit the growth of other pathogenic organisms or microbiological spoilage agents, having the status of GRAS (Generally Recognized as Safe). These bacterial species are entirely natural. Therefore, they provide a useful “green” benefit to food product labeling [8]. Bacteriocin-producing strains have gained considerable interest in recent years. They are considered one of the most promising alternatives to antibiotics for use as protective cultures.

Enterococci are ubiquitous microorganisms found in the gastrointestinal tracts of humans and animals and in water, soil, plants, and food. These microorganisms produce bacteriocins known as enterocins [9], which exhibit an inhibition spectrum against taxonomically close bacteria and even those with a broad spectrum of action, inhibiting a wide range of bacteria, including the emergent *C. perfringens* [10,11]. Using enterococci as potential probiotic strains or protective cultures can be an excellent alternative to antibiotic use in poultry farming [12].

However, in recent years, the use of enterococci in the food industry has been debated because of their implications for opportunistic infections and their potential acquisition of antimicrobial resistance and virulence genes [9]. Therefore, developing new enterococcal probiotics requires a strict safety assessment to select the truly harmless enterococcal strains for safe applications [13].

This study aimed to isolate and characterize *Enterococcu*s strains of poultry origin that might exhibit antimicrobial activity against *C. perfringens* and other relevant microorganisms.

## 2. Results

### 2.1. Enterococcus Sampling and Identification

Sixty enterococcus strains were isolated from poultry meat samples collected from local markets in La Rioja, Spain. These strains were identified using MALDI-TOF mass spectrometry as *E. faecium* (*n* = 33), *E. faecalis* (*n* = 19), *E. gallinarum* (*n* = 5), *E. casseliflavus* (*n* = 1), *E. durans* (*n* = 1), and *E. avium* (*n* = 1). These isolates were combined with another 191 *Enterococcus*, previously obtained from poultry (in Spain and Tunisia), and with five bacteriocin-producing (BP+) *Enterococcus* from other origins, to develop the entire collection of 256 *Enterococcus* used to detect and characterize the BP+ isolates.

### 2.2. Screening of Enterococci for Antimicrobial, Specifically Anti-C. perfringens Activity

**Figure 1:**
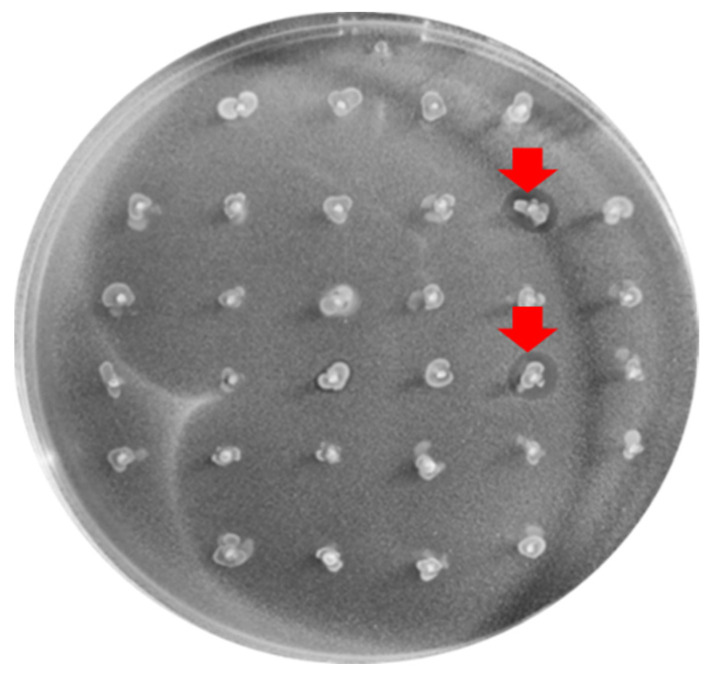
Inhibition halos (marked with the red arrow) produced by 2 of the BP+ enterococci tested against the *C. perfringens* X2967 indicator strain.

In total, 32 of the 256 enterococci tested (12.84%) demonstrated antimicrobial activity against *C. perfringens* X2967 using the “spot on the lawn” method. These strains belonged to the species *E. faecium* (*n* = 11), *E. gallinarum* (*n* = 9), *E. faecalis* (*n* = 8), *E. durans* (*n* = 3), and *E. casseliflavus* (*n* = 1). Among them, 27 (84,37%) were active against *Listeria monocytogenes, Micrococcus luteus*, and *Streptococcus suis* (Table 1). One *Enterococcus* strain alone showed antimicrobial activity against methicillin-susceptible *Staphylococcus aureus* (MSSA) and methicillin-resistant *S. aureus* (MRSA). None of the tested strains showed inhibitory activity against gram-negative bacteria (*Escherichia coli*, *Salmonella enterica, Yersinia enterocolitica*, and *Pseudomonas aeruginosa*). Figure 1 shows the inhibition halo against *C. perfringens* X2967 produced by two of the thirty-two BP+ strains.

### 2.3. Effects of the Supernatants of BP+ Enterococci on C. perfringens Isolates

**Figure 2:**
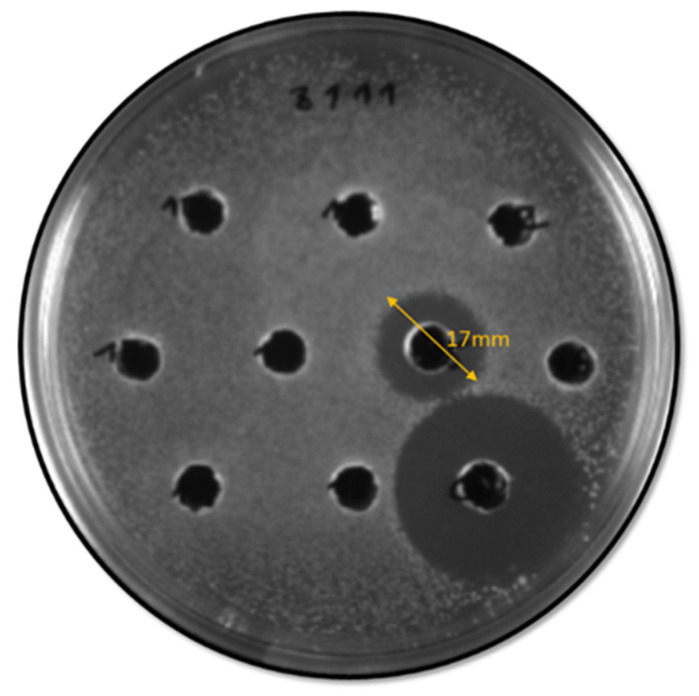
Inhibition halo of the *E. faecium* strain, X3179, against one of the 20 *C. perfringens* isolates. The bigger halo corresponds to the activity of nisin, used as a control.

The supernatants of the 32 BP+ enterococci were tested against a collection of 20 *C. perfringens* isolates of poultry origin. The antimicrobial activity was detected in 18 concentrated supernatants against at least one of the *C. perfringens* strains. Nevertheless, antimicrobial activity was observed in six of the heated supernatants (HS) and non-heated supernatants (NHS) (Figure 2, Table 2), corresponding to four *E. faecium* and two *E. durans* isolates. In general, the inhibitory activities of the HS and NHS were similar; both inhibited the growth of 2–8 strains of the 20 *C. perfringens* tested. The concentrated supernatants showed a broad spectrum of inhibition against 2–20 *C. perfringens* isolates (Table 2). The remaining 14 supernatants, either HS, NHS, or concentrated supernatants, did not show any inhibitory activity.

Supernatant activity could only be quantified for the *E. faecalis* X3198 and *E. faecium* X3179 strains (16 AU/mL).

### 2.4. Phenotypic and Genotypic Characterization of the Selected BP+ Enterococci

For a complete genome analysis, 12 BP+ enterococci were selected based on their antimicrobial activity detected using the previously described methods. Five *E. faecium* and two *E. faecalis* of poultry origin were selected, as well as five *E. durans* of milk and camel milk origin, chosen as the BP+ controls.

#### 2.4.1. Bacteriocinome

Structural genes encoding for bacteriocins were detected in 12 BP+ strains (Table 3). The structural genes for enterocins P and Enterocin L50 A/B were detected in all five *E. durans* isolates, and the genes for bac 32 were also observed in three of them. Genes encoding enterocin A and enterocin B were detected in all the *E. faecium* strains; two of these strains carried the genes encoding enterocin NKR-5-3-A/D/Z. Moreover, the genes encoding enterocin SE-K4 and staphylococcin C55a/b were identified in two *E. faecalis* strains.

#### 2.4.2. Antibiotic Resistance phenotype and resistome

Five of the twelve selected BP+ enterococci (41.7%) were susceptible to the nine antibiotics tested, all of them from the species *E. durans*. The remaining strains were resistant to at least one of the antibiotics tested. The most frequent resistance was against ciprofloxacin (58.3%), followed by tetracycline (25.0%), erythromycin (25.0%), penicillin (16.7%), chloramphenicol (8.3%), high-level streptomycin (8.3%), and high-level gentamicin (8.3%). In addition, all the isolates showed susceptibility to vancomycin and linezolid.

Genes encoding antibiotic resistance were detected in all 12 BP+ strains (Table 4), although only five (three *E. faecium* and two *E. faecalis* isolates) had genes for acquired-type resistance. The mutations associated with resistance phenotypes for beta-lactams (*pbp5*) and fluoroquinolones (*gyrA* and *parC*) were detected only in *E. faecium* isolates (Supplementary Material).

#### 2.4.3. Virulence

- Gelatinase activity and hemolysis

Among the 12 selected BP+ enterococci, only *E. faecalis* X3198 was positive for gelatinase activity, and all the strains showed gamma hemolysis.

- Virulome

Among the 12 BP+ enterococcal strains, virulence genes were detected *in E. faecium* and *E. faecalis* but not in *E. durans* (Table 5).

#### 2.4.4. Plasmidome

The replicon plasmids identified in the selected enterococci are listed in Table 6. All of the *E. durans* strains carried *RepA_N, Inc18*, and *Rep3* or *Rep1* plasmidic replicons. Both *E. faecalis* strains carried the type *Rep trans*. Moreover, most of th*e faecium* strains carried at least three different types of plasmidic replicons.

#### 2.4.5. Genetic Lineages

**Figure 3:**
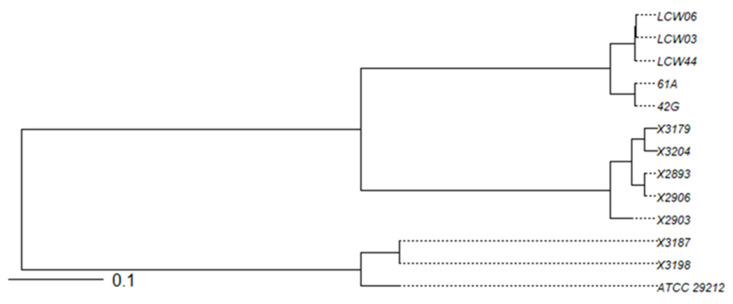
Phylogenetic tree based on the average nucleotide identity (ANI) of the 12 BP+ enterococci. The reference strain, ATCC 29212, was also included.

Multi-locus sequence typing (MLST) of the two *E. faecalis* and five *E. faecium* strains yielded the following results: (a) the two *E. faecalis* strains were typed as ST397; (b) the five *E. faecium* strains showed four different sequence types, with two isolates typed as ST722, one isolate typed as ST784, and the remaining two with an unknown ST (Table 5, Figure 3).

## 3. Discussion

### 3.1. Screening for BP+ Enterococci

A total of 32 of the 256 enterococci tested (12.84%) showed antimicrobial activity against the *C. perfringens* X2967 strain, as determined using the “spot on the lawn” method; however, among these, only 18 supernatants of the BP+ strains were active against the collection of 20 *C. perfringens* isolates used as indicators. The inhibitory activities of these supernatants were attributed to the *Enterococcus*-derived enterocins [14]. The absence of inhibitory activity in the supernatants obtained from the strains showing inhibition using the spot-on-the-lawn method may be explained by the fact that bacteriocins sometimes remain attached to the cell wall and are not released in the supernatant. Furthermore, the production of bacteriocins is commonly mediated by quorum sensing [15]; hence, we detected 14 strains as BP+ via the spot-on-the-lawn method (in which the producer and the indicator strains are confronted) but without activity in their supernatants (the extract produced without previous exposure to the indicator bacteria) [16].

### 3.2. Phenotypic and Genotypic Characteristics of the BP+ Enterococci

According to their antimicrobial activity, 12 BP+ enterococci were selected for further characterization.

#### 3.2.1. Bacteriocinome

The structural genes for enterocins P and Enterocin L50A/B were detected in all five *E. durans* isolates. Enterocin P (entP) was first detected in an *E. faecium* strain isolated from a dry-fermented sausage [17], showing activity against gram-positive pathogenic bacteria such as *C. perfringens, L. monocytogenes*, and *S. aureus*. Enterocin P is chromosomally encoded [18,19]; however, other studies have detected *entP* genes in the plasmid location [20]. Enterocin P and L50A/B have been detected in different enterococcal species [21]. This study is the first study to detect Enterocin P in *E. durans*.

Enterocin L50A/B was first detected in an *E. faecium* L50 strain isolated from Spanish fermented sausage [22]. Enterocin L50A/B consists of two peptides, L50A and L50B, which synergistically promote their antimicrobial activity. The strain *E. faecium* L50 has also been shown to produce enterocins Q and P at different temperatures [18,23]. Enterocin L50 A/B exhibits a broad spectrum of antimicrobial activities, including inhibition of *Enterococcus* spp., *Lactobacillus* spp., *Lactococcus lactis*, *Pediococcus pentosaceus*, *L. monocytogenes*, *S. aureus*, *B. cereus*, *C. botulinum*, *Streptococcus pneumoniae*, *S. mitis*, *S. oralis*, *S. parasanguis*, *S. agalactiae*, and *C. perfringens*. Other enterocins, such as enterocins 7A/7B and MR10A/10B, share a strong homology with enterocin L50 A/B [21]. 

Enterocin bac 32 was identified in three of our five *E. durans* strains. This peptide was firstly detected in a vancomycin-resistant clinical *E. faecium* VRE200 strain, exhibiting activity against *Enterococcus* spp [24]. Although this bacteriocin has not been extensively studied, it seems to be identical to enterocin IT [25].

The strain *E. durans* 61A has been previously described, and durancin 61A and enterocins L50A and L50B were identified using mass spectrometry [26,27]. However, the genetic determinants for these bacteriocins were not detected in strain 61A using whole genome sequencing (WGS) in our study; instead, enterocin P was detected. Duracin 61A is not in the anti-SMASH and BAGEL4 databases (we used data from the NCBI and NCBI plus UniProt, respectively), whose genetic determinants have yet to be described. In contrast, enterocin P might not have been detected in other studies, as it is a temperature-regulated bacteriocin that is synthesized optimally at 37–47 °C [23].

Genes encoding enterocin A and enterocin B were detected in all of our five *E. faecium* strains, two of which also carried the genes encoding Enterocin NKR-5-3-A/D/Z.

Enterocin A was first identified in 1996 [28] and is produced by several strains of *E. faecium*—CTC492, T136, and P21—isolated from Spanish sausage; BFE900 from black olives; DPC 1146, WHE 81, and EFM01 from dairy products; and the N5 strain of “nuka”, a Japanese rice paste. Enterocin A shows activity against *Enterococcus* spp., *Lactobacillus* spp*., Pediococcus* spp., and *L. monocytogenes* [10]. However, its activity has not been tested against clostridial species. Enterocin A is usually co-produced with enterocin B, which is produced by *E. faecium* T136 isolated from Spanish fermented sausages [29]. Enterocin B shows antimicrobial activity against gram-positive bacteria, such as *L. monocytogenes*, *Propionibacterium* spp., *C. sporogens*, and *C. tyrobutyricum* [29]. When enterocin A and enterocin B are co-produced, they form a heterodimer, and studies have demonstrated its potential anti-bacterial and anti-biofilm activities against *S. aureus*, *Acinetobacter baumannii*, *L. monocytogenes,* and *E. coli* [30].

The genetic determinants for enterocin NKR-5-3-A/B/C/D/Z were detected in two of our *E. faecium* strains. These enterocins have been purified and studied previously [31]. NKR-5-3-A (identical to brochocin A) and NKR-5-3-Z are class IIb bacteriocins and exhibit synergistic antimicrobial activity. NKR-3-5-B is a novel circular bacteriocin belonging to class IIc bacteriocins with a broad spectrum of antimicrobial activities against *Bacillus* spp., *Enterococcus* spp., and gram-negative bacteria (*E. coli* and *Salmonella*). NKR-5-3-C is a class IIa bacteriocin with strong antimicrobial activity against *L. monocytogenes*. NKR-5-3-D, a class IId bacteriocin, has a weak antimicrobial activity but can be produced even under unfavorable conditions [32,33]. NKR-5-3-A, D, and Z variant genes were detected in the *two E. faecium* strains. The genetic determinants of enterocins NKR-5-3-A/C/D/Z are closely located in a gene cluster (13 kb long) and include specific bacteriocin biosynthetic genes, such as an ABC transporter gene (*enkT*), two immunity-related genes (*enkIaz* and *enkIc*), a response regulator (*enkR*), and a histidine protein kinase (*enkK*). This gene cluster is essential for the biosynthesis and regulation of NKR-5-3 enterocins [34].

Genes encoding enterocin SE-K4 and staphylococcin C55a/b were identified in the two *E. faecalis* strains in this study. Enterocin SE-K4 was first identified in *E. faecalis* K-4 isolated from grass silage [35]; it grows at 43–45 °C and exhibits antimicrobial activities against *E. faecium*, *E. faecalis*, *B. subtilis*, *C. beijerinckii*, and *L. monocytogenes*. This enterocin has a high degree of homology to bacteriocin 31 and T8/43 [10]. Staphylococcin C55a/b was originally found to be produced by *S. aureus* C55 [36], consisting of three distinct peptide components termed staphylococcins C55a, C55b, and C55g. Staphylococcins C55a and C55b (lantibiotic components) acted synergistically against *S. aureus* and *M. luteus* [36]. It is a plasmid-encoded bacteriocin [37]; thus, the plasmid transfer between the producer, *Staphylococcus*, and the *E. faecalis* strains could account for the presence of the genetic determinants of this bacteriocin.

#### 3.2.2. BP+ *Enterococcus* Resistance Phenotype and Resistome 

Five of the twelve BP+ enterococci, all from *E. durans* isolates, were susceptible to the nine antibiotics tested. The remaining strains showed resistance to at least one of the antibiotics. Generally, the enterococci of poultry origin have more resistance genes than those of other origins (camel and camel milk). The only gene discovered in *the E. durans* strains of milk origin was *aac(6′)-Iih*, which is intrinsically present in *E durans* [38,39]. Antibiotics are commonly used in poultry farming, leading to the development of acquired resistance mechanisms in poultry-derived strains. 

The genus *Enterococcus* is characterized by its intrinsic resistance to several antibiotics and ability to acquire new resistance mechanisms [40]. Enterococci are naturally resistant to semisynthetic penicillins (a reduced susceptibility), aminoglycosides (in low levels), vancomycin (at a low level and only in the species *E. gallinarum* and *E. casseliflavus/E. flavescens*, which are carriers of *vanC* genes), to lincosamides, polymyxins, and streptogramins (the species *E. faecalis*) [41]. In addition, *E. faecium* carries some intrinsic genes, such as *msrC* and *aac(6′)-Ii,* whereas *E. durans* harbors the gene *aac(6′)-Iih* [38,39]. Antibiotic resistance can occur either through the acquisition of genetic elements containing the resistance genes or via DNA mutations (mostly in genes encoding antibiotic targets), which are favored when there is a selective antibiotic pressure [40].

Among the acquired resistance genes detected in the *E. faecium* and *E. faecalis* strains, the genes associated with erythromycin [*erm(B)*], chloramphenicol [*fexB* and *cat*]*,* tetracycline [*tet(M)* and *tet(L)*], streptomycin [*str*], gentamicin and tobramycin [*aac(6′)-aph(2″)*]*,* and linezolid resistance (*poxtA)* have been reported. Vancomycin resistance genes have not been reported [42].

*E. faecium* strains X2893 and X2906 carry only chromosomal and intrinsic resistance genes (*msr(C)* and *aac(6′)-Ii)*, which are non-transferable; therefore, these strains are excellent candidates for use as potential protective cultures.

Specific mutations in the *pbp5* and *gyrA*/*parC* genes are associated with resistance to beta-lactams and fluoroquinolones, respectively [43,44,45]. Different mutations in the *pbp5*, *gyrA,* and *par*C genes have been detected in our strains, although, in most cases, with an unknown resistance phenotype associated.

#### 3.2.3. Virulence of BP+ Enterococci

Different virulence factors are involved in the attachment to host cells and extracellular matrix proteins (AS, Esp, Hyl, and EfaA), macrophage resistance (AS), and cell and tissue damage (Cyl and GelE) [46,47]. Thus, although enterococci are commensal bacteria found in the intestine, they can still cause infections. Therefore, the Food and Drug Administration (FDA) has not yet assigned them to the GRAS category. Genes encoding these virulence factors are located in conjugative plasmids (*agg*, *cyl*, or *hyl*), in the chromosome (*gelE* or *fsr*), or in regions of the chromosome called pathogenic islands (*esp* and *cyl*) [48,49].

In the 12 enterococcal strains, virulence genes were detected in *E. faecium* and *E. faecalis* but not in *E. durans*. *E. faecalis* has already been described as more virulent than other species [50]. Fifteen virulence genes were detected in both *E. faecalis* strains. However, the presence of these genes is not always related to the virulence potential, as they are sometimes silenced and not associated with the phenotype [49]. Both strains carried the *gelE* gene, which is associated with gelatinase activity, but only strain X3198 was positive for gelatinase activity.

All the *E. faecium* strains carried the functional collagen adhesin gene, *acm*, which plays an essential role in colonization by binding to collagen type I, with less affinity to collagen type IV [51]. As these *E. faecium* strains did not carry other virulence factors, the presence of *acm* might be positive, as it could facilitate the colonization of this beneficial strain. Nevertheless, as mentioned before, the presence of a virulence gene does not always indicate that it is being expressed [49]. Therefore, further studies must uncover whether *acm* is, in fact, expressed as a virulence factor. 

#### 3.2.4. Plasmidome of the BP+ Enterococci

Ten of the BP+ enterococci harbored at least one plasmid. Interestingly, strains X2893 and X2906 did not present any mobile genetic elements, which, along with the other characteristics, makes them good candidates for potential protective cultures [52].

## 4. Materials and Methods

### 4.1. Enterococcus Sampling and Identification

In total, 251 enterococcal isolates of poultry origin were used in this study: (a) 60 isolates were collected during this study from poultry carcass samples obtained from different supermarkets and butchers in the La Rioja Region (Spain), the isolates recovered in the Slanetz–Bartley agar (OXOID); (b) 166 isolates were previously obtained from poultry carcasses at the slaughterhouses’ level in Tunisia; (c) 25 poultry isolates were obtained from the University of La Rioja’s collection (Spain). Additionally, 5 BP+ enterococci of other origins (2 isolates from cow milk and 3 from camel milk) were obtained from the University of LAVAL’s strain collection (Canada). 

### 4.2. Screening for Anti-C. perfringens Activity Using the “Spot on the Lawn” Method

The antimicrobial activity of the 256 *Enterococcus* isolates against the indicator strain, *C. perfringens* X2967 (a clinical strain obtained from the Hospital San Pedro, Logroño, Spain), was analyzed using the “spot-on-the-lawn” method [53]. The active isolates were identified as BP+. Briefly, a fresh culture of *C. perfringens* strain X2967 was suspended in brain–heart infusion broth (BHI) (turbidity 0.5 MacFarland). Subsequently, 10 µL of this indicator microorganism solution was added to tubes containing 5 mL of semi-solid melted tryptic soy broth (TSB) and supplemented with 0.7% agar and 0.3% yeast extract. Finally, the semi-solid TSB medium with the indicator microorganism was poured onto tryptic soy agar plates (TSA). Once the plates were dried, the enterococcal microorganisms were sting-seeded, and the plates were incubated at 37 °C for 24 h under strict, anaerobic conditions.

Strains that showed inhibitory activity against *C. perfringens* strain X2967 were tested against other relevant pathogens and multidrug-resistant (MDR) bacteria using the same test. This panel included *E. casseliflavus* C1232, *E. gallinarum* C2310*, E. faecium* C2321, *E. faecalis* C410, *E. durans* C1433*, E. hirae* C1436, MSSA C411, MRSA C1570, *M. luteus* C157*, L. monocytogenes* C137, *S. suis* C2058, *E. coli* C408, *S. enterica* C660, *Y. enterocolitica* X3080, and *P. aeruginosa* X3282. A blood agar plate was used for *S. suis* testing. All strains used as indicator bacteria came from the University of La Rioja’s collection.

### 4.3. Screening for Anti-C. perfringens Activity Using the Agar Diffusion Method

NHS and HS extracts were prepared from *Enterococcus* isolates showing inhibitory activity in the spot-on-the-lawn assay. These supernatants were tested against a collection of 20 *C. perfringens* isolates using the previously described agar diffusion method [54], with nisin as a positive control. The *C. perfringens* isolates were collected from the NE of poultry origin (University of Laval, Quebec, QC, Canada).

To prepare the NHS, enterococci were inoculated in 10 mL of TSB in sterile tubes and were incubated overnight at 37 °C. Then, the culture medium was centrifuged at 5000× *g* rpm for 5 min and filtrated using 0.20 µm filters. Next, a fraction of this supernatant was heated at 100 °C for 15 min and used as the HS. For the concentrated supernatants, the culture cell media were concentrated 10 times using a Speed Vac (Thermo Scientific Savant, Asheville, NC, United States) after centrifugation.

For the agar well-diffusion method, *C. perfringens* was cultured in a reinforced clostridial medium (RCM) (Himedia, Kennett Square, PA, USA) supplemented with 10% agar. The plates were incubated overnight at 37 ºC under strict, anaerobic conditions. 

### 4.4. Anti-C. perfringens Activity Determination Using Microtitration Assay

A microtitration assay was performed to determine the total activity (AU/mL) of the active supernatant of BP+ enterococci against the *C. perfringens* ATCC 13124 strain, as described previously [55,56]. The BHI was used as the growth medium for *C. perfringens* and was added to the wells, with a final bacterial concentration of ~10^5^ CFU/well. The microplate was incubated for 24 h at 37 °C under strict, anaerobic conditions. After incubation, the optical density was measured at 595 nm using a microplate reader (Infinite M200, Tecan, Männedorf, Switzerland) to determine the number of wells in which inhibition occurred.

The following formula was used to calculate the total arbitrary activity:

$$Activity\;\left(\frac{AU}{mL}\right)=2^{n*\frac{1000}{25}}=2^{\left(n+3\right)}$$

where 2 is the dilution factor, n is the number of inhibition wells, 1000 is the factor for reporting the result per mL, and 125 is the volume of the solution tested in microliters.

### 4.5. Characterization of BP+ Enterococci

Twelve BP+ enterococci were chosen for further characterization based on their antimicrobial activity against *C. perfringens* strains.

#### 4.5.1. Susceptibility to Antibiotics

The susceptibility of BP+ enterococci to nine antibiotics was tested using the disk diffusion method according to the Clinical and Laboratory Standard Institute (CLSI) guidelines (2020) [57]. The antibiotics tested were as follows (disk charge): penicillin (10 units), tetracycline (30 µg), erythromycin (15 µg), chloramphenicol (30 µg), linezolid (30 µg), high-level gentamicin (120 µg), high-level streptomycin (300 µg), vancomycin (30 µg), and ciprofloxacin (5 µg). Strains were then identified as susceptible (S), resistant (R), or intermediate (I) using the protocol interpretation guidelines [57].

#### 4.5.2. Gelatinase Activity and Hemolysis

The gelatinase activity and hemolytic capacity of BP+ enterococci strains were determined as reported previously [58], using TSA supplemented with 3% skim milk and blood agar, respectively.

#### 4.5.3. Whole Genome Sequencing (WGS) Analysis

DNA from BP+ enterococci was extracted using a DNeasy Blood & Tissue Kit (QIAGEN, Hilden, Germany), following the manufacturer’s instructions for gram-positive bacteria. The DNA was subjected to WGS using an Illumina sequencing system at the Hospital Center of University Laval (CHUL). Data were analyzed using the following programs; fastp for trimming and quality check of the trimming [59], SPAdes for the assembly [60], QUAS for checking the assembled quality [61], and prokka for annotation [62]. Anti-SMASH 6.0 [63] and BAGEL4 [64] were used to detect genes encoding bacteriocins. ResFinder 4.1 [65,66,67] was used to detect genes associated with antibiotic resistance and mutations in the *pbps*, *parC*, and *gyrA* genes. VirulenceFinder 2.0 was used to detect virulence factors [67,68,69] and PlasmidFinder 2.1 for plasmid detection [67,70]. Multi-locus sequence typing (MLST) was performed using MLST 2.0 [71,72,73,74,75,76]. Representation in the phylogenetic tree was performed using R version 4.2.1 [77], and the phylogenetic distances were calculated using the average nucleotide identity (ANI) method.

## 5. Conclusions

Among the 12 enterococci that showed inhibitory activity against *C. perfringens*, the strains *E. faecium* X2893 and X2906 seem to be the most promising candidates for use as protective cultures in poultry farming. Both strains belong to the sequence type ST722 and harbor enterocin A and Enterocin B genetic determinants. These strains also do not have acquired resistance genes, do not carry plasmids, and only carry the *acm* gene, which is implicated in host colonization and might be a desirable feature for protective strains. Both are gelatinase-negative and gamma-hemolytic.

The strains derived from other origins (milk and camel milk) and belonging to the species *E. durans* might be also good candidates as protective cultures, as they do not harbor any virulence factors or resistance genes, and they produce bacteriocins. However, these strains carry more than one plasmid and have not been isolated from poultry.

Concluding, *E. faecium* X2893 and X2906 showed potential to be considered in further studies as protective cultures in poultry farming, a promising alternative to antibiotic use in this sector.

## Figures and Tables

**Table 1:** Antimicrobial activity of the 32 bacteriocin producer (BP+) enterococci against *C. perfringens* X2967 and other relevant indicator bacteria^a^, as detected by the “spot on the lawn” assay.

		Number of BP+ Strains with Activity against the Indicator Strain					
		*E. faecium* (*n* = 11)	*E. gallinarum* (*n* = 9)	*E. faecalis* (*n* = 8)	*E. durans* (*n* = 3)	*E. casseliflavus* (*n* = 1)	Total
Indicator strains ^a^	*C. perfringens* (X2967)	11	9	8	3	1	32
	*E. hirae* (C1436)	11	1	7	3	1	23
	*E. durans* (C1433)	11	5	2	3	1	22
	*E. casseliflavus* (C1232)	8	3	6	3	-	20
	*E. faecium* (C2321)	10	3	3	3	-	19
	*E. faecalis* (C410)	11	1	3	3	-	18
	*E. gallinarum* (C2310)	9	3	3	3	-	18
	*L. monocytogenes* (C137)	8	4	4	3	-	19
	*M. luteus* (C157)	1	1	7	3	1	13
	*S. suis* (C2058)	2	-	6	3	1	7
	MRSA ^b^ (C411)	-	1	-	-	-	1
	MSSA ^b^ (C1570)	-	1	-	-	-	1

^a^ None of the isolates showed antimicrobial activity against *E. coli* (C408), *S. enterica* (C660), *Y. enterocolitica* (X3080), or *P. aeruginosa* (X3282). ^b^ Abbreviation: MRSA: methicillin-resistant *S. aureus*; MSSA: methicillin-susceptible *S. aureus.*

**Table 2:** The number of *C. perfringens* isolates to which the supernatants of 18 BP+ enterococci present antimicrobial activity in their supernatants.

BP+ Strain	Origin	Species	Number of *C. perfringens* (of 20 Tested) Inhibited by the Antimicrobial Activity of the Extracts of BP+ Strains		
			Non-Heated Supernatant	Heated Supernatant	Concentrated Supernatant
C1446	Poultry	*E. gallinarum*	-^a^	-	11
X2829	Poultry	*E. faecium*	2	2	12
X3036	Poultry	*E. gallinarum*	-	-	2
X3179	Poultry	*E. faecium*	4	4	18
X2903	Poultry	*E. faecium*	-	-	8
X2947	Poultry	*E. faecium*	-	-	4
X2956	Poultry	*E. faecium*	-	-	3
X2960	Poultry	*E. faecium*	-	-	4
X3187	Poultry	*E. faecalis*	-	-	1
X3220	Poultry	*E. faecium*	-	-	1
X3198	Poultry	*E. faecalis*	-	-	1
X3204	Poultry	*E. faecium*	2	2	16
X2906	Poultry	*E. faecium*	2	2	8
61A	Cow milk	*E. durans*	4	3	20
42G	Cow milk	*E. durans*	8	5	18
LCW03	Camel milk	*E. durans*	-	-	18
LCW44	Camel milk	*E. durans*	-	-	16
LCW06	Camel milk	*E. durans*	-	-	16

^a^ The symbol -: no antimicrobial activity was detected against any of the 20 *C. perfringens* isolates tested as the indicator bacteria.

**Table 3:** Putative enterocins detected by WGS in the 12 selected BP+ enterococci.

Strain	Species	Putative Enterocins
42G	*E. durans*	Enterocin P, Enterocin L50 A/B
61A	*E. durans*	Enterocin P, Enterocin L50 A/B
LCW03	*E. durans*	Enterocin P, Enterocin L50 A/B, Bacteriocin 32
LCW06	*E. durans*	Enterocin P, Enterocin L50 A/B, Bacteriocin 32
LCW44	*E. durans*	Enterocin P, Enterocin L50 A/B, Bacteriocin 32
X2893	*E. faecium*	Enterocin A, Enterocin B
X2903	*E. faecium*	Enterocins NKR-5-3A; Enterocin NKR-5-3D, Enterocin NKR-3-5-3-Z
X2906	*E. faecium*	Enterocin A, Enterocin B
X3179	*E. faecium*	Enterocin A, Enterocin B, Enterocin NKR-5-3A, Enterocin NKR-5-3D, Enterocin NKR-5-3-Z
X3204	*E. faecium*	Enterocin A, Enterocin B
X3198	*E. faecalis*	Ent SE-K4, Staphylococcin C55a/b
X3187	*E. faecalis*	Ent SE-K4, Staphylococcin C55a/b

**Table 4:** Antibiotic resistance phenotype and genotype of the BP+ enterococci.

Strain	Species	Origin	Antibiotic Resistance Phenotype	Antibiotic Resistance Genotype	
				Intrinsic Mechanisms	Acquired Mechanisms
X2893	*E. faecium*	Poultry	CIP	*msr(C), aac(6′)-Ii*	-
X3179	*E. faecium*		CIP, E	*msr(C), aac(6′)-Ii*	*erm(B)*
X2903	*E. faecium*		CIP, E, P, C	*msr(C), aac(6′)-Ii*	*erm(B), fexB, poxtA*
X3187	*E. faecalis*		CIP, E, P, TE, CN, S	*Isa(A)*	*erm(B), aac(6′)-aph(2″), tet(M)*
X3198	*E. faecalis*		CIP, TE	*Isa(A)*	*erm(B), aac(6′)-aph(2″), tet(M)*
X3204	*E. faecium*		CIP, TE	*msr(C), aac(6′)-Ii*	*str, tet(M), tet(L), cat*
X2906	*E. faecium*		CIP	*msr(C), aac(6′)-Ii*	-
61A	*E. durans*	Other	Susceptible	*aac(6′)-Iih*	-
42G	*E. durans*		Susceptible	*aac(6′)-Iih*	-
LCW03	*E. durans*		Susceptible	*aac(6′)-Iih*	-
LCW44	*E. durans*		Susceptible	*aac(6′)-Iih*	-
LCW06	*E. durans*		Susceptible	*aac(6′)-Iih*	-

**Table 5:** Virulence genes and sequence types detected in the BP+ *E. faecalis* and *E. faecium* isolates of poultry origin by WGS.

Strain	Species	Virulence Genes	Sequence Type
X3187	*E. faecalis*	*ElrA, SrtA, ace, cCF10, cOB1, cad, camE, ebpC, efaAfs, fsrB, gelE, hylA, hylB*, and *tpx*	ST397
X3198	*E. faecalis*	*ElrA, SrtA, ace, cCF10, cOB1, cad, camE, ebpC, efaAfs, fsrB, gelE, hylA, hylB*, and *tpx*	ST397
X2893	*E. faecium*	*acm*	ST722
X2903	*E. faecium*	*acm*	New allelic combination: *adk-1, atpA-2, ddl-7, gdh-57, gyd-1, pstS-80, purK-6*
X2906	*E. faecium*	*acm*	ST722
X3179	*E. faecium*	*acm*	New allelic combination: *adk-1, atpA-2, ddl-7, gdh-76, gdh-2, gyd-1, pstS-1, purK-3*
X3204	*E. faecium*	*acm*	ST784

**Table 6:** Plasmidome of the 12 BP+ enterococci detected by WGS.

Strain	Species	Type	Replicon Plasmid
X2893	*E. faecium*	-	-
X3179	*E. faecium*	Rep3	*rep29*
		Inc18	*rep1, rep2*
		RepA_N	*repUS15*
X2903	*E. faecium*	Rep3	*rep29*
		Rep1	*repUS58*
		Inc18	*rep1*
		Rep_trans	*rep14a*
		RepA_N	*repUS15*
X3187	*E. faecalis*	Rep_trans	*repUS43*
X3198	*E. faecalis*	Rep_trans	*repUS43*
X3204	*E. faecium*	RepA_N	*repUS15*
		Inc18	*rep1*
		Rep1	*rep22*
X2906	*E. faecium*	-	*-*
61A	*E. durans*	RepA_N	*repUS15*
		Rep1	*repUS64*
		Inc18	*rep1, rep2*
42G	*E. durans*	RepA_N	*repUS15*
		Rep1	*repUS64*
		Inc18	*rep1, rep2*
LCW03	*E. durans*	Rep3	*rep18a*
		Inc18	*rep1, rep2*
		RepA_N	*repUS15*
LCW44	*E. durans*	Inc18	*rep1, rep2*
		Rep3	*rep18a*
		RepA_N	*repUS15*
LCW06	*E. durans*	Inc18	*rep1, rep2*
		Rep3	*rep18a*
		RepA_N	*repUS15*

## Supplementary Materials

The following supporting information can be downloaded at: https://www.mdpi.com/article/10.3390/antibiotics12020231/s1, File S1: Supplemented material on mutations in *pbp5*, *gyrA* and *parC* found in the 12 BP+ enterococci analyzed by WGS.
